# The Upregulation of OCT4 in Acidic Extracellular pH is Associated with Gemcitabine Resistance in Cholangiocarcinoma Cell Lines

**DOI:** 10.31557/APJCP.2019.20.9.2745

**Published:** 2019

**Authors:** Phatchareeporn Choodetwattana, Siriporn Proungvitaya, Patcharee Jearanaikoon, Temduang Limpaiboon

**Affiliations:** 1 *Centre for Research and Development of Medical Diagnostic Laboratories, Faculty of Associated Medical Sciences, *; 2 *Cholangiocarcinoma Research Institute, Khon Kaen University, Khon Kaen, Thailand. *

**Keywords:** Chemoresistance, tumor microenvironment, octamer-binding transcription factor 4, liver cancer

## Abstract

**Background:**

Cholangiocarcinoma (CCA), although is an uncommon liver cancer originating from bile duct epithelial cells, is one of the top 10 most fatal cancers. Chemoresistance is an unmet need always found in CCA patients. Tumor microenvironment conditions such as hypoxia, nutrient starvation and acidic extracellular pH play critical roles in chemoresistance and cancer progression. However, the effect of acidic extracellular pH on cellular response and chemoresistance in CCA has not been studied.

**Methods:**

Human CCA cell lines (KKU-M213, KKU-M055 and KKU-100) were cultured under acidic (pH 6.5) or non-acidic (pH 7.4) condition and were used for gene expression, doubling time and cytotoxicity assay.

**Results:**

The acidic extracellular pH (pH 6.5) significantly increased doubling times of CCA cell lines compared with non-acidic condition (pH 7.4). Interestingly, extracellular acid condition induced gemcitabine resistance in CCA cell lines. We showed that Octamer-binding transcription factor 4 (Oct4) was upregulated in these cell lines under extracellular acid condition.

**Conclusion:**

Our findings demonstrate that CCA cells can adapt to survive in acidic environment after which chemoresistance has been developed. Oct4 may be a key transcriptional regulator which mediates chemoresistance in response to acidic extracellular pH.

## Introduction

Cholangiocarcinoma (CCA) is the most common liver cancer found in Northeast Thailand where the incidence of liver fluke (*Opisthorchis viverrini*) infection is high. Surgical resection is an effective curative treatment for CCA. Although adjuvant chemotherapy has significantly improved overall survival in CCA patients, the responsiveness is relatively low with a partial response approximately 10-20% and median overall survival is only 4 months (Bhudhisawasdi et al., 2012; Luvira et al., 2016). 

The tumor microenvironment has increasingly appeared as a key player in the development of chemoresistance and tumor progression in the past decade (Yuan et al., 2016; Senthebane et al., 2017). Several studies have demonstrated that the extracellular pH of solid tumors is acidic (Fukamachi et al., 2010; Zhang et al., 2010; Estrella et al., 2013). Lactic acid produced by anaerobic glycolysis in hypoxic condition seems to be the main cause of acidic extracellular pH, which is an environmental stressor being toxic to many cells, including tumors. Such acidic microenvironment exerts a selective pressure, if tumors have successfully adapted to this condition, they develop more aggressive behavior contributing to chemoresistance and tumor progression (Ibrahim-Hashim and Estrella, 2019). Som et al., (2016) demonstrated that acidic microenvironment in solid tumors induced the expression of octamer-binding transcription factor 4 (Oct4) in fibroblasts and other stromal cells. The Oct4 protein, a transcription factor encoded by the Pou5f1 gene also known as Oct-3, Oct-3/4, Otf3 or NF-A3, belongs to the POU (Pit, Oct, Unc) family of DNA binding-proteins. It binds to the octamer motif ATGCAAAT within the promoter or enhancer regions of target genes to regulate their expression. The expression of Oct4 is associated with pluripotent properties of embryonic stem cells, it is absolutely required for controlling early stages of mammalian embryogenesis (Zeineddin et al., 2014).

However, the effect of acidosis on CCA cell behavior, gene expression and chemotherapeutic response remains unknown. Accordingly, in this study, we aimed to elucidate the behavioral change in CCA cell lines cultured under acidic environment. In this condition, we show that CCA cells slow down cell division, decrease gemcitabine sensitivity and upregulate Oct4 gene expression. Our findings indicate that CCA cells enable to survive in acidic environmental stress and this adaptation may have the effect at least in part on chemotherapeutic treatment. The expression of Oct4 implies some of the stem cell-like phenotypes which may mediate chemoresistance in CCA.

## Materials and Methods

*Cell lines and cell culture*

Human CCA cell lines (KKU-M213, KKU-M055 and KKU-100) established in the Cholangiocarcinoma Research Institute, Khon Kaen, Thailand were used in the study (Sripa et al., 2005). Cells were cultured in Ham’s F12 Nutrient Mixture (Gibco-BRL, Ontario, Canada) supplemented with 10% fetal bovine serum and 1% penicillin/streptomycin (Gibco-BRL) at 37°C in a 5% CO_2_ atmosphere under acidic (pH 6.5) or non-acidic (pH 7.4) condition by which the culture medium was changed every day. Acidic medium was prepared by adding 12 M HCl to Ham’s F-12 until the desired pH was obtained. After incubation for 5 days, cells were harvested by trypsinization using 0.5% trypsin-EDTA (Gibco-BRL) and used for gene expression, doubling time and cytotoxicity assay.

*RNA isolation and reverse transcription-polymerase chain reaction (RT-PCR)*

Total RNA was isolated from all cell lines using TRIzol^®^ reagent (Invitrogen, Carlsbad, CA, USA). The cDNA was synthesized by reverse transcription of total RNA using ImProm-II^TM^ Reverse Transcription System (Promega Corporation, Madison, WI, USA) according to the manufacturer’s protocols. The primer sequences were as follows; Oct4a: forward 5’-GGTTGAGTAGTCCCTTCGCAAGC-3’; reverse 5’-CTTAGCCAGGTCCGAGGATCAAC-3’ and glyceraldehyde 3-phosphate dehydrogenase (GAPDH): forward 5’-ATGTTCGTCATGGGTGTGAA-3’; reverse 5’-AGAGGCAGGGATGATGTTCT-3’. The PCR reaction was carried out using a Rotor-Gene Q (Qiagen, South San Francisco, CA, USA). The 50 µL of PCR reaction consisted of 1x PCR buffer (67 mM Tris, pH 8.4, 16.6 mM ammonium sulfate and 0.1% Tween20), 0.2 µM of each primer, 200 µM of each dNTP, 50 ng of cDNA, 1.5 µM SYTO^®^9 (Invitrogen), 2 mM MgCl_2 _and 5 units of Taq DNA polymerase. The cycling stage was performed as following steps: initial denaturation at 90°C for 10 min, 40 cycles of denaturation at 90°C for 20 sec, and annealing and extension at 62°C for 20 sec. Relative gene expression was analyzed by the comparative Ct method (2^-ΔΔCt^).

*Doubling time assay*

The doubling time of CCA cell lines was performed using Sulforhodamine B (SRB) assay. Briefly, cells were fixed with 10% (W/V) trichloroacetic acid, stained with SRB for 30 min, and washed repeatedly with 1% (V/V) acetic acid to remove excess dye. The protein-bound dye was dissolved in 10 mM Tris base solution and determined for the absorbance at 510 nm using a microplate reader (Tecan Ltd., Reading, UK). The doubling time was determined using an on-line calculator (http://www.doubling-time.com/compute.php).

*Cytotoxicity assay*

Cells with density of 2x10^3^ were seeded in triplicate in a flat-bottom 96-well plate and allowed to grow for 24 h. Then, 100 µL of medium containing different concentrations of gemcitabine were added to each well to get a final concentration of 10, 20, 40, 80, 160 and 320 nM, respectively. After 72 h, cell viability was performed using SRB assay. The percentage of cell viability was calculated using the following formula: (mean ODsample)/ (mean ODday0) x 100. 

*Statistical analysis*

All experiments were performed independently three times. The data were expressed as mean ± standard deviation (SD) and analyzed using SPSS 17.0 software (SPSS Inc., Chicago, IL, USA). The differences in doubling time, cell viability and relative gene expression between acidic and non-acidic condition were analyzed using Student’s t-test. P ≤ 0.05 was considered statistically significant.

## Results

*Cells cultured in acidic extracellular pH showed longer doubling time*

**Figure 1:**
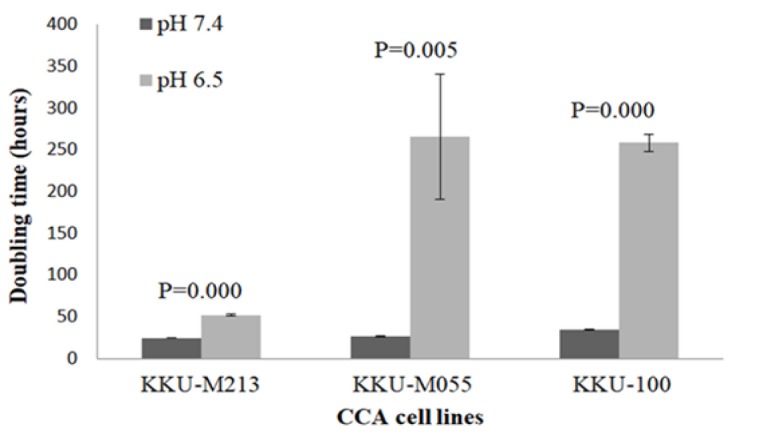
The Population Doubling Time of CCA Cell Lines under Non-Acidic (pH 7.4) and Acidic (pH 6.5) Conditions

The population doubling time of KKU-M213, KKU-M055 and KKU-100 was determined after being cultured for 5 days under acidic (pH 6.5) and non-acidic (pH 7.4) conditions. It was found that acidic extracellular pH significantly increased doubling time in all CCA cell lines when compared to cell grown in pH 7.4 (Figure 1), indicating the reduction of cell growth in acidic environment.

**Figure 2:**
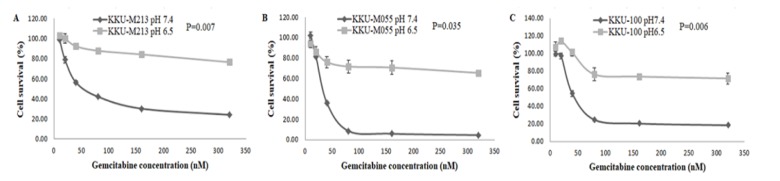
Effects of Gemcitabine on Cell Survival. CCA cell lines were treated with various concentrations of gemcitabine for 72 h then cell viability was performed using SRB assay. (A) KKU-M213, (B) KKU-M055, (C) KKU-100

**Figure 3:**
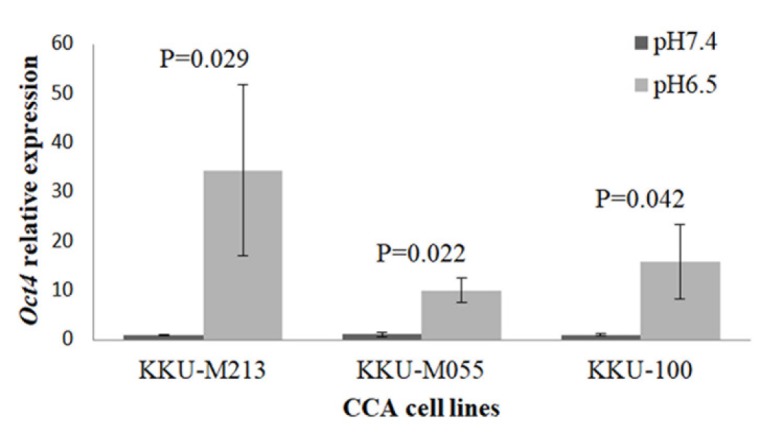
Relative mRNA Expression of Oct4 in CCA Cell Lines

*Acidic extracellular pH caused gemcitabine resistance in CCA cell lines*

After culture for 5 days under acidic (pH 6.5) and non-acidic (pH 7.4) conditions, cells were treated with different concentrations of gemcitabine for 72 h and determined for cell viability. We found that cells grown in pH 6.5 remained viable and resistant to gemcitabine even at high concentration (320 nM) while cells cultured in pH 7.4 were killed and sensitive to gemcitabine (Figure 2). Our finding suggested that acidic extracellular pH had an effect on gemcitabine resistance in CCA cell lines.

*Acidic extracellular pH induced the upregulation of Oct4*

It has been reported that acidic extracellular pH could induce the expression of Oct4 in fibroblast and stromal cells in tumor models (Som et al., 2016). Our study showed that Oct4 was significantly highly upregulated in CCA grown in acidic pH when compared to those cultured in non-acidic condition (Figure 3).

## Discussion

Tumor microenvironment such as hypoxia, nutrient starvation and acidic extracellular pH play critical roles in tumor growth, invasion and metastasis, as well as anti-cancer drug resistance (Estrella et al., 2013; Ibrahim-Hashim and Estrella, 2019). The extracellular pH of tumor tissues is often acidic, which lactic acid produced by anaerobic and aerobic glycolysis (Warburg effect) is the major cause and CO_2_ production *via* the pentose phosphate pathway is an alternative cause of acidic microenvironment (Helmlinger et al., 2002; Vander Heiden et al., 2009). The extracellular pH of solid tumors becomes acidic with the pH range of 6.4-6.9 (Gerweck and Seetharaman, 1996). Many lines of evidence indicate the important effect of acidic microenvironment on both cancer and stromal cells as well as their behavior (Kato et al., 2013; Som et al., 2016). We have shown that all CCA cell lines cultured under acidic pH had significantly longer doubling time than those grown in non-acidic condition. Our study was consistent with the study of Kondo et al., (2017), which performed the effect of low pH, hypoxia and nutrient starvation culture conditions on cell growth and adhesion of PANC-1 and AsPC-1 pancreatic cancer cells. They showed that acidic extracellular pH (pH 6.8) significantly reduced cell growth and adhesion of PANC-1 and AsPC-1 compared with control (pH 7.4). Moreover, acidic pH decreased cell growth compared with hypoxia and nutrient starvation in PANC-1 and AsPC-1 suggesting that acidic pH triggered different cellular responses from hypoxia and nutrient starvation. 

Adjuvant therapy such as chemotherapy and radiotherapy is given to the patients after surgery to prevent recurrence of the disease. Chemoresistance is an unmet need which causes recurrence, dissemination and death in cancer patients. The molecular mechanisms of chemoresistance include transporter pumps, oncogenes, tumor suppressor gene, mitochondrial alteration, DNA repair, autophagy, epithelial-mesenchymal transition (EMT), cancer stemness, and exosome (Zheng, 2017). Moreover, the pH in the tumor microenvironment can affect the cytotoxicity of anti-cancer drugs. Anti-cancer drugs mainly target the rapidly proliferating cancer cells. Therefore, slow-growing cells have a trend to develop chemoresistance. This study demonstrated the high percentage of cell viability in the presence of gemcitabine under acidic condition. Our findings indicate the low efficiency of gemcitabine even at high concentration, which cannot kill slowly dividing cancer cells under acidic microenvironment resulting in chemoresistance. It has been shown that lactic acid can also contribute to tumor radioresistance, due to its antioxidant properties (Sattler et al., 2010). 

Our study revealed that Oct4 was upregulated in response to acidic extracellular pH, which was different from the study of Kondo et al., (2017). They showed that extracellular acidity activates the upregulation of sterol regulatory element-binding protein-2 (SREBP2), a transcriptional regulator of cholesterol biosynthetic enzymes, by which acyl-CoA synthetase short-chain family member 2 (ACSS2), a direct SREBP2 target, promotes tumor growth and progression in pancreatic cell lines. They also showed that transcriptional regulators identified under hypoxia are hypoxia-inducible factor 1A (HIF1A) and HIF2A, and under nutrient starvation are activating transcription factor 4 (ATF4) and forkhead box O3 (FOXO3). In this study, acidic extracellular pH could facilitate cellular reprogramming, as indicated by increased Oct4 expression. The expression of Oct4 implies some of the stem cell-like phenotypes which may mediate chemoresistance in CCA. Therefore, the use of anti-Oct4 as a targeted therapy may be an innovative regime for effective treatment of CCA.

In summary, we demonstrate the effect of acidic extracellular pH on cellular behavior of CCA cell lines including reduction of cell growth, increase of gemcitabine resistance and upregulation of Oct4. Our findings may shed light on the important role of tumor microenvironment, in particular acidic extracellular pH, on the development of chemoresistance in CCA, in which the manipulation of Oct4 must be scrutinized for treatment efficacy.
